# Concerns about the Burden of Proof studies

**DOI:** 10.1038/s41591-023-02294-8

**Published:** 2023-04-14

**Authors:** Andrea J. Glenn, Xiao Gu, Frank B. Hu, Molin Wang, Walter C. Willett

**Affiliations:** 1 Department of Nutrition, Harvard T.H. Chan School of Public Health, Boston, MA USA; 2 Department of Nutritional Sciences, Temerty Faculty of Medicine, University of Toronto, Toronto, Ontario Canada; 3 Toronto 3D Knowledge Synthesis and Clinical Trials Unit, Clinical Nutrition and Risk Factor Modification Centre, St. Michael’s Hospital, Toronto, Ontario Canada; 4 Channing Division of Network Medicine, Department of Medicine, Brigham and Women’s Hospital and Harvard Medical School, Boston, MA USA; 5 Department of Epidemiology, Harvard T.H. Chan School of Public Health, Boston, MA USA; 6 Department of Biostatistics, Harvard T.H. Chan School of Public Health, Boston, MA USA

**Keywords:** Epidemiology, Epidemiology

arising from P. Zheng et al. *Nature Medicine* 10.1038/s41591-022-01973-2 (2022).

arising from H. Lescinsky et al. *Nature Medicine* 10.1038/s41591-022-01968-z (2022).

arising from J. D. Stanaway et al. *Nature Medicine* 10.1038/s41591-022-01970-5 (2022).

arising from C. Razo et al. *Nature Medicine* 10.1038/s41591-022-01974-1 (2022).

arising from X. Dai et al. *Nature Medicine* 10.1038/s41591-022-01978-x (2022).

We read with interest the Burden of Proof (BoP) studies^1–5^ in which the authors conducted meta-analyses of epidemiological studies to provide an overall conservative quantitative assessment for several important public health questions. For ease of interpretation, they transformed the overall assessment into a star rating (1–5 stars). Examples include five stars for smoking and lung cancer, two stars for low vegetable intake and ischemic heart disease (IHD) and two stars for unprocessed red meat and type 2 diabetes (T2D), colorectal cancer and IHD^6^. They used this same method to assign just three stars to smoking in relation to IHD and one or two stars to other well-established relationships^1–4^. However, we believe there are serious methodological issues with their meta-analyses^5^; the star rating of evidence strength is overly simplistic and could cast doubt on existing recommendations and policies intended to prevent chronic disease and treat illnesses.

In the BoP analyses, the estimated uncertainty intervals (UIs) were several times wider than the 95% confidence intervals (CIs) generated by existing methods that account for heterogeneity among methods^7^. As an example of the BoP analyses^1^, for unprocessed red meat and T2D 15 studies were included in the meta-analysis, many of which were large and without evidence of small study bias. With what the authors described as a conventional analysis, there is a highly statistically significant and approximately linear positive association and the relative risk for 100 g per day of red meat intake versus no consumption was approximately 1.24. However, due to an approximately 2.5-fold inflation of the width of the conventional CIs due to the BoP methodology, their lower 95% boundary included no association, which was then used to rate evidence and two stars were given. Using the well-established Greenland and Longnecker method^8^ we estimated the linear association for each study included in Lescinsky et al.^1^ and then used a standard random effects meta-analysis method (taking into account the extra uncertainty due to estimating the between-study variance) to calculate a summary association relating unprocessed red meat and T2D. A robust positive dose–response is seen (*P* < 0.001) and the 95% CIs in our analysis (relative risk = 1.24, 95% CI = 1.12–1.36) is only modestly wider than that from the conventional fixed effects model. Our analysis, which takes into account between-study heterogeneity in associations, suggests that the UIs provided by the BoP studies are extreme. This issue is further illustrated by their rating of only three stars for smoking and IHD based on meta-analyses of 60 studies, similar to the rating for smoking and lower back pain (only six studies)^4^. The relationship of smoking to risk of IHD, primarily acute myocardial infarction, is extremely strong and approximately linear with relative risks over 5 for high-intensity smoking; this risk has been recognized as convincing for over four decades by regular reports of the U.S. Surgeon General^9^. At least six mechanistic pathways have been documented^9^. On the other hand, the association between smoking and lower back pain lacks clear evidence of causation.

The UIs from the BoP meta-analyses are intended to account for other sources of heterogeneity beyond between-study heterogeneity in mean effects. The authors conducted bias assessment of individual studies, including exposure and outcome measurements, controlled for confounding and selection bias. In the meta-analysis of red meat and six chronic disease outcomes, none of the bias adjustments were statistically significant^1^. In addition, there is minimal evidence of publication bias or influence of outlier observations from small studies. These findings suggest that the authors’ assumption about between-study heterogeneity is untenable and their methods that lead to drastically inflated UIs beyond conventional random effects models are not justified.

Meta-analyses of studies are commonly used to synthesize published evidence but they cannot overcome variation in the original studies because of different definitions of exposure and outcome, follow-up time, data analyses and covariate adjustment. We believe that a better approach is to obtain individual-level data from all original studies and use standard definitions of the primary exposure and covariates and then model the relationship between exposures and outcomes. This approach is now widely used in nutritional epidemiological studies, providing more robust and informative estimates; these analyses can additionally examine subgroup effects. When using individual-level data to summarize studies, substantive unexplained heterogeneity has been uncommon^10^. Another assumption made by the BoP authors is that the dose–response relationship is not log-linear. However, previous studies of red meat consumption in relation to risks of diabetes^11^ and cardiovascular disease^12^ have concluded that within the range of population intakes, there was no substantive deviation from linearity. We believe that a more appropriate approach is to meta-analyze the estimates from continuous exposures from each study, which can enhance statistical power, reduce between-study heterogeneity and facilitate the interpretation of the data. Still, a critical examination of the studies that contribute most to the conclusions is desirable to evaluate the assessment of exposure and outcome, control of confounding, potential for reverse causation and other aspects of study design.

Furthermore, when evaluating the relationship between one dietary component, such as red meat, and health outcomes, alternative foods such as poultry, fish or plant protein must be considered because a person’s long-term energy intake is tightly regulated within narrow limits without a substantial change in weight or physical activity^13^. The choice of counterfactuals can make a major difference and substitution analyses are now routinely part of epidemiological analyses^13^, which could not be addressed in the BoP papers as they require primary data.

Beyond the methodological issues raised here, the totality of the evidence, rather than only epidemiological studies, is critical to consider when drawing conclusions about causality and providing recommendations. The ideal randomized trial in humans is rarely feasible but the totality of evidence could include randomized controlled trials of intermediate risk factors for diseases, salient animal studies and plausible biological mechanisms. For example, in randomized controlled trials, compared with plant protein sources, red meat (high in saturated fat and cholesterol and low in polyunsaturated fat) increases low-density lipoprotein cholesterol^14^, which in turn increases the risk of IHD. Even without prospective cohort evidence linking red meat and IHD, on the basis of these data one could extrapolate that eating large amounts of red meat might be harmful for heart disease. This type of evidence is part of the Bradford Hill criteria^15^ for evaluating epidemiological evidence and determining in expert reviews whether there is a sufficiently strong basis for translation to recommendations and policies^15^.

Considering the methodological and conceptual problems we raise with the new meta-analysis methods, the star rating system for the BoP does not seem justifiable. The authors note that the star ratings were designed for policy-makers and individuals to make decisions about their own risk. However, many people may not be aware of the crucial subtleties in studies of diet and other complex environmental exposures and the need to consider other forms of evidence. Although the authors suggest that the precautionary principle may apply when there is some evidence of harm, the BoP estimates imply that these estimates are so uncertain that strong public health recommendations and policies should not be made based on so-called weak evidence. We agree with the authors that further research in many areas is desirable. However, because the CIs in the BoP methodology tend to cover the findings from almost all existing studies, including extreme studies close to the null hypothesis, new studies showing clear associations may have little effect on the BoP conclusions. It is our view that BoP studies are misleading and the star rating system is too simplistic. We believe that reviews by national and international bodies that consider the full range of evidence on these topics are likely to provide a better basis for personal and policy decisions.
